# Emotions under Discussion: Gender, Status and Communication in Online Collaboration

**DOI:** 10.1371/journal.pone.0104880

**Published:** 2014-08-20

**Authors:** Daniela Iosub, David Laniado, Carlos Castillo, Mayo Fuster Morell, Andreas Kaltenbrunner

**Affiliations:** 1 Cologne Graduate School, University of Cologne, Cologne, Germany; 2 Social Media Research Group, Barcelona Media Foundation, Barcelona, Spain; 3 Qatar Computing Research Institute, Doha, Qatar; 4 Berkman Center, Cambridge, Massachusetts, United States of America; Middlesex University London, United Kingdom

## Abstract

**Background:**

Despite the undisputed role of emotions in teamwork, not much is known about the make-up of emotions in online collaboration. Publicly available repositories of collaboration data, such as Wikipedia editor discussions, now enable the large-scale study of affect and dialogue in peer production.

**Methods:**

We investigate the established Wikipedia community and focus on how emotion and dialogue differ depending on the status, gender, and the communication network of the 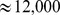 editors who have written at least 100 comments on the English Wikipedia's article talk pages. Emotions are quantified using a word-based approach comparing the results of two predefined lexicon-based methods: LIWC and SentiStrength.

**Principal Findings:**

We find that administrators maintain a rather neutral, impersonal tone, while regular editors are more emotional and relationship-oriented, that is, they use language to form and maintain connections to other editors. A persistent gender difference is that female contributors communicate in a manner that promotes social affiliation and emotional connection more than male editors, irrespective of their status in the community. Female regular editors are the most relationship-oriented, whereas male administrators are the least relationship-focused. Finally, emotional and linguistic homophily is prevalent: editors tend to interact with other editors having similar emotional styles (e.g., editors expressing more anger connect more with one another).

**Conclusions/Significance:**

Emotional expression and linguistic style in online collaboration differ substantially depending on the contributors' gender and status, and on the communication network. This should be taken into account when analyzing collaborative success, and may prove insightful to communities facing gender gap and stagnation in contributor acquisition and participation levels.

## Introduction

Emotions are the glue of human societies [1] (cf. [2], p.27) and their significant influence on human behavior is undisputed. Online collaborative communities, as increasingly important social spaces for teamwork and self-expression, make no exception and are permeated by emotions [3]. Yet, little is known about the emotional “ecosystem” of online collaborative endeavors, and our work contributes to this knowledge gap.

As field of study we choose the English Wikipedia, one of the largest peer-production communities, which provides an excellent case scenario, given its size and importance. The conversations in the Wikipedia discussion pages are especially valuable. These pages represent arenas of cooperation and conflict between users (to whom we will refer to as *editors* from now on) with the goal of improving encyclopedic content.

Conversation is essential for coordination in such spaces [4], and therefore facilitates fruitful collaboration and successful content creation. Like in any other human collaborative experience, communication also triggers emotions and breeds particular emotional environments that may influence teamwork in the short and long run.

To analyze the emotional expression and communication style used in Wikipedia discussions we utilize two established word-counting measures and differentiate according to status and gender. Since each lexicon highlights different aspects of emotion and language, their concurrent use provides us with a rich understanding of editor interactions.

Indisputably, differences in status also reflect differences in language use [5], and the communication context in which emotions play out becomes interesting to investigate. Consequently, our first research question analyzes how emotional expression and communication style in online collaboration differ according to status. We expect to find differences in emotional expression according to the status of Wikipedia editors since research on emotion in social structures suggests that emotional expression is a significant marker of status in the social hierarchy [2]. Analyzing emotions in this context may prove insightful considering the slow-down in editor growth from 2006 onwards [6].

Our analyses bring insight into the emotional profile of higher status contributors which is important since the emotions of individuals higher in rank are contagious for followers and can impact the performance of the entire social structure [7]. These contributors bear therefore the responsibility to channel their emotions towards constructive ends for the community.

Similarly, gender has been found to explain diverse facets of language and emotion in offline settings [8], hence we expect Wikipedia male editors to display a different communication and emotional mode compared to female ones. The second focus of our research is, therefore, on how the emotional expression and communication style of editors depend on gender. The differences are worth exploring, considering that the gender gap (i.e., a strong inequality in the gender distributions of the participants) is a serious source of concern for this collaborative community [9].

In our discussion of the first two research questions we refer to a crucial concept in the leadership literature – relationship-oriented communication [10], a type of communication focused on establishing and maintaining social ties [11]. We expect to find evidence that Wikipedia editors exhibit features of this kind of speech, and we analyze online discussions to uncover status and gender differences in its use.

Finally, the network of editors and messages in Wikipedia can provide interesting evidence for phenomena such as “emotional congruence” or “emotional homophily” in a peer-production context. We investigate them as third and fourth research questions. More exactly, we pursue a brief analysis of Wikipedia's interaction networks based on messages and replies exchanged between editors to examine how editors' emotions relate to the emotions of the editors they reply to during interaction (emotional congruence), and how the emotions of editors are related to those of the editors they interact more frequently with (emotional homophily). These phenomena have been observed in online fora [12] and blogs [13]; they refer to the similarity of comment-reply pairs regarding expressed emotion – emotionally-loaded dialogue is followed by replies with higher emotional content, whereas neutrality is met with neutrality – and to the principle of “birds of a feather flock together” – users tend to interact with others expressing similar emotions.

## Background

The following section presents a short overview of related research in diverse fields such as computer-science, psychology and communication, structured according to the research focus.

### Communication on Wikipedia

The largest human encyclopedia ever written, Wikipedia, is one of the most prominent examples of successful online collaboration to date. In fact, considering the thousands of failed online collaboration efforts [14], its size and success are quite miraculous. This noteworthy performance has motivated a flurry of research activity [15] on topics ranging from leadership behaviors to motivations to contribute.

Communication is quintessential to Wikipedia. The complex interaction system allows for persistent social interaction among participants, which facilitates the emergence, maintenance and continuous redefinition of social structures and collective goals [16]. Editors frequently discuss changes to articles on *article talk pages*, while *user talk* pages (“personal” pages) resemble a personal wall and function as a public mail inbox [17]. Both types of pages are used for interaction, but the conversation in article talk pages is article-focused, quite “formalized and policy driven” [18] and meant to ensure article quality, while discussion on personal talk pages is editor-focused [19]. In light of the different emphasis of Wikipedia talk pages, our research sheds light on how this translates into individual patterns of emotion and communication.

### Emotions and peer-production

A recent contribution on emotions in the online collaborative world is presented in [20]. Similar to the present paper, the study presents an investigation of emotions in Wikipedia depending on gender and status. The analysis is based on the ANEW (Affective Norms for English Words) lexicon and shows that women express more positive emotion than men, while higher-status editors are more positive compared to lower-status ones. We draw heavily on the research presented in [20] by using it as a starting point to compare and contrast our results regarding emotions. On the other hand, we diverge significantly from it, since our investigation allows for a richer and more fine-grained analysis at both the emotion and communication level. In particular, the word-counting measures we use facilitate the understanding of emotion within the communication context.

Earlier work on the relationship between emotions and online contribution behavior can be traced back to Joyce and Kraut [21], who identified no relationship between receiving positive feedback and the likelihood of posting again in a public newsgroup. Simply receiving feedback was enough to increase contribution behavior, irrespective of the valence (i.e., positive and negative affect) of the response.

On the other hand, subsequent research found clear evidence that feedback valence impacts participation behavior. For example, Cheshire and Antin [22] investigated the effect of response on contribution behavior, and found that positive feedback such as gratitude (e.g., receiving automatic thank you replies) increased the users' number of solved puzzles.

Similarly, Wang, Kraut and Levine [23] reported that emotional support in the form of caring messages increased the commitment to an online health support group more so than informational support. In fact, informational support alone was associated with a greater risk of drop-off from the community, whereas the combination of emotional and informational support was the most effective for continued participation in the community.

A study especially relevant to this research was undertaken by Zhu, Kraut and Kittur [24] on the effect of leadership styles on contribution behavior in Wikipedia. The authors focused on the outcome of task-oriented and relationship-oriented communication. Task-oriented communication is characterized by assertive, directive and instrumental speech such as directive statements, information provision, as well as critical evaluation of contributions [25]. On the other hand, relationship-oriented communication is affiliative and includes expression of support, agreement, and acknowledgment of others' contributions [26]. The authors found that relationship-oriented messages increased the probability of subsequent edits. On the contrary, negative feedback decreased members' contributions greatly. Messages coming from higher-status were more influential compared to those coming from lower-status members.

A recent study by Kucuktunc et al. [27] investigated emotions in a large-scale Q&A community depending on factors such as gender, age or experience in the community. Their findings suggest that women are more emotional and express more positive emotion compared to men, while more experienced users give increasingly neutral answers.

There is also an interesting stream of literature on the network properties of emotions. For example, Chee [12] finds in an online health forum that those who communicate often have similar emotion levels, while Thelwall [13] reports the same conclusion for MySpace. This is consistent with either a homophily hypothesis (“Birds of a feather flock together”) or with an emotional contagion explanation. The present work briefly investigates this phenomenon from a network analysis perspective in a novel, peer-production context, without aiming at disentangling the two competing explanations.

Finally, with regard to Wikipedia, previous studies have investigated mixing patterns in this community from different perspectives, to assess whether there is a tendency of editors to interact with similar others (assortativity). Diversity according to experience and volume of activity [17], [28] has been observed as a distinguishing characteristic of interactions in the community: expert editors tend to interact preferentially with newbies and less active editors, and vice-versa. On the other hand, assortative mixing patterns have been observed with respect to other markers of identity, such as gender: females tend to interact more with other females, and males with other males [20]. From a social identity perspective, a recent study highlighted that Wikipedians tend to communicate more with others supporting the same political party in personal talk pages, while no preference was observed in article talk pages [29]. Emotional expression and style of editors have also been found to drive their interactions, with a preference for communication with editors having a similar style [20]; here we deepen this analysis by taking into account also discrete emotions and linguistic styles of editors.

### Conversational and emotional markers of status and gender

Human language contains markers of status, and social hierarchy can be understood through and mapped by analyzing language [30], [31]. Hence, in this work we will compare the power relation in Wikipedia on several dimensions of written expression, and expect to find salient differences, also with regard to emotional aspects.

#### Status

First, previous literature on communication differences between the powerful versus the weak indicates that those lower in status use more tentative speech as a signal of insecurity [32], such as *maybe, could, should*. We expect to find such differences between high-status Wikipedia editors (whom we will refer to as *administrators* or *admins*) and regular editors.

Moreover, there is much interest in the literature with regard to the significance of first-person singular pronouns. The evidence is mixed, however: whereas the higher use of first-person active pronouns (e.g., “I”) can commonsensically be viewed as a signal of power or empowerment [32], a number of text analyses with the LIWC lexicon [33]–[35] find the opposite, i.e. heightened use of “I” is associated with the lower status of the writer. This self-focus has been interpreted as a strategy to draw attention to the less-powered self, in an attempt to highlight one's importance and merits. Taking gender into account, while an earlier study by Mulac, Bradac and Gibbons [36] finds evidence for men's use of first singular pronouns, more recent studies such as Mehl and Pennebaker [37] and Newman and colleagues [38] find that females are more self-focused. We study the use of “I” pronouns for the Wikipedia community, and propose several explanations for the results.

Despite being a showcase for open collaboration and peer-production, Wikipedia is not an open-source setting with a flat hierarchy. In fact, some authors contend that a strong motivation to contribute to Wikipedia is gaining and exercising power in the community [39], [40]. Higher-status Wikipedia editors are called *administrators* or *admins*. They are a special category of Wikipedia editors who generally assume responsibility in the community, which is associated with privileges compared to regular editors. For example, they are allowed to delete pages or block other editors [41].

There is little research on the emotional and language differences depending on status in Wikipedia. Panciera et al. [42] find that gaining status in Wikipedia leads to an increased formalization of speech. For example, higher-status editors refer more often to Wikipedia policies during discussion. On the other hand, they maintain a positive attitude, while less-experienced editors express more negative emotion [43]. We add to this literature by providing a much more detailed account of the differences in emotion and language use between administrators and regular editors.

#### Gender

This paper also examines gender differences in terms of emotional and linguistic expression. Previous literature finds a robust marker of gender – the positivity of language. Women use more positive language, and particularly so when engaged in conversation. Mehl and Pennebaker [37] found these results in a naturalistic conversation setting, and Newman et al. [38] found that women express more positive emotion when interacting with others, but not in other contexts, e.g., essays or stream of consciousness (a stream of consciousness writing task asks participants to write down thoughts and feelings). Additionally, Kivran-Swaine and colleagues [44] found that in Twitter women disclose more positive emotion than men, and especially so when interacting with other women. Given previous research, we expect Wikipedian women to use more positive language compared to male editors.

Furthermore, women are more likely than men to practice relationship-oriented speech to positively engage with others. A meta-analysis conducted by Leaper and Ayres [45] finds that women use significantly more affiliative speech than men. However, the difference is statistically significant only for conversations on non-personal topics, self-disclosures, and deliberations, as well as for same-gender groups (but not for mixed-gender groups). Men, on the other hand, use significantly more task-oriented, assertive speech than women. Again, the effect is significant for same-gender groups, and for discussions of non-personal topics or deliberations. Since the interaction in Wikipedia is generally on non-personal topics, yet takes place in a mixed-gender setting, we do not have a precise theoretical expectation concerning gender differences in relationship-oriented speech.

Previous literature is not clear on whether there is a gender advantage in the expression of negative emotion. While some authors find that women express more negative emotions than men [46], [47], others (e.g. [38]) report the opposite.

Finally, we investigate gender differences in the expression of anger. Previous research indicates that societal expectations discourage women from showing anger [48], [49], and that men have an advantage in the use of anger words [37]. While Newman and colleagues [38] do not find evidence for this for all types of text, they do so for conversations, suggesting that the gender difference in anger expression is activated particularly during social interactions. Consequently, we expect that women will use fewer anger words compared to men.

Women are severely under-represented in the Wikipedia community, leading to an important gender gap. The participation rate for female editors is a subject of contention, ranging from a mere 9% [9] to 16.1% [50]. Its low level has been attributed to women's conflict aversion and sensitivity to criticism, as well as to a lack of confidence in their expertise and contribution value [51]. Given that 22% of female editors have reported disagreeable interactions and receiving inappropriate messages [9], our investigation of emotions and dialogue could prove insightful for the gender gap issue.

## Materials and Methods

In this section we describe the dataset consisting of Wikipedia discussions on article and personal talk pages, as well as the methodology used for the analysis of emotions and language.

### Dataset

For comparison purposes we make use of the same dataset used in [20]. Only the conversation history of the most active editors in discussion pages was retained from a complete snapshot of the English Wikipedia [52]. This consists of the 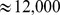 editors who have written at least 100 comments during discussions on article talk pages; since not all editors have a personal page, this reduced the dataset to 11637 editors for the personal talk pages. Table 1 reports the basic statistics of the data.

**Table 1:** Dataset characteristics.

Articles	3 210 039	
Articles with talk page (ATP)	871 485	(27.1%)
Editors who comment articles	350 958	
Editors with ≥100 comments on ATP	12 231	(3.5%)
Total comments in ATP	11 041 246	
Comments containing ANEW words	7 414 411	(67.2%)
Comments made by editors with ≥100 comments on ATP	5 480 544	(49.6%)
Comments made by these editors used for sentiment analysis (containing ANEW words)	3 649 297	(33.3%)

The dataset contains information on editors' status (administrator versus regular editor) and gender, which allows comparison across different editor groups. While information on editor status is available through the Wikipedia API, collecting gender information is less straightforward and can prove challenging. In this case gender identification was possible using a combination of methods, ranging from using Wikipedia's API to crowdsourcing the gender identification task to Crowdflower (see [20] for more details). Table 2 summarizes the editor sample classified according to status and gender.

**Table 2:** Editors with at least 100 comments by status and gender.

	Non-admins	Admins	Total
Males	1 087	1 526	2 613
Females	68	97	165
Unknown	6 850	2 603	9 453
Total	8 005	4 226	12 231

### Sentiment analysis

We measure the emotional content of comments in article talk pages and user talk pages with a lexicon-based method consisting of two established word-counting measures - the Linguistic Inquiry and Word Count (LIWC) [53] and SentiStrength [54]. The simultaneous use of several types of lexicons allows us to: (i) cross-validate the results of our and previous analyses, given that emotional valence is a common characteristic of lexicons; (ii) offer a rich understanding of interactions, since each lexicon highlights different aspects of emotion and language.

Not least, we aim to compare and contrast our results with those obtained using ANEW (Affective Norms for English Words) and shown in [20]. For a meaningful comparison, we first selected only those comments from the discussions in the articles and personal talk pages for which at least one ANEW word was found. This decision was done to restrict the possible influence of the article and discussion topics, as reported in [20], on the results. This sampling strategy resulted in a database of more than 7.4 million comments for the article talk pages, and around 3 million comments for the personal pages, which were then used for the computation of the LIWC and SentiStrength scores restricting us on the subset of these comments written by our set of editors.

There are slight differences across the lexicons with regard to the definition of valence for each comment:

- LIWC gives a positive and a negative score representing the percentage of positive(negative) words in a comment [53],
- SentiStrength provides a positive and a negative score based on the most positive(negative) sentence in a comment [54],
- while ANEW provides a single score on a scale from 1 (extremely negative) to 9 (extremely positive) [55].

Regardless of these differences, LIWC and SentiStrength results by and large validate and confirm the ANEW findings of [20] regarding valence, as we will explain in detail in the Results section. Moreover, our lexicon comparison allows us to bring additional understanding to the Wikipedian landscape, particularly with regard to gender- and status-related differences in language use. The following paragraphs describe in more detail the characteristics of the three lexicons, as well as the procedural steps we applied for the text- and sentiment analysis of Wikipedia content.

### LIWC

The Linguistic Inquiry and Word Count (LIWC) was developed by the psychologists Pennebaker, Booth and Francis in the early 90s to automatize psychological analysis of written expression, and is now the preferred automated sentiment detection method in psychology, while also gaining ground in the computer sciences. Moreover, the LIWC lexicon [53] allows us to identify language differences between Wikipedia editor groups that go beyond emotion expression.

We aggregated all comments of a single editor (comments posted by editors on article talk pages) in one file, which was then analyzed with LIWC. For the personal pages we analyzed with LIWC the entire content of an editor's talk page, and discriminated between messages received and written by an editor on her own talk page.

LIWC provides two scores for basic emotion: positive valence and negative valence. Positive (negative) valence in LIWC is defined as the percentage of positive (negative) words [53] in a text, in this case as the percentage from the total number of words written by an editor during her Wikipedia activity (which takes values between 0 and 100).

We also analyzed the comments with respect to the many different groups of measures that LIWC provides – e.g. relationship-orientation, temporal orientation or certainty of the written expression. This allowed us to bring additional insight to the differences in emotion and language use between the Wikipedia editor groups.

These additional LIWC measures, including personal pronouns such as “I” or “you”, several discrete measures of emotions (e.g., anger, anxiety and sadness) or social words, are derived in a similar fashion to the LIWC basic emotions, as the percentage of words from a given category compared to the total number of words written by an editor during her Wikipedia activity. The LIWC dictionary for anger contains words such as *annoyed*, *argh* or *bastard*; the one for anxiety, *stressed*, *terrifying*, *uneasy*, etc.; while the measure for sadness includes *agony*, *depressed* or *grief*. The group of certainty measures refers to the use of tentative words (*anyhow*, *depends*, *doubt*), certainty words such as *guaranteed* or *obviously*, as well as filler words, e.g., *oh well*, *i mean*. Temporal orientation refers to the use of verbs in past, present or future tense. Finally, the dictionary for social words is composed of words referring to family, friends and humans in general, e.g., *reply*, *daughter*, *baby*. The percentage of social words together with the percentages of personal pronouns of different types define the group of relationship-orientation metrics. An overview of the LIWC measures with dictionary sizes and further examples can be found in Table 3. We also performed a lexicon validation for the categories relationship-orientation, certainty and anger, which lead accuracy levels situated around 0.70. Details about the results for these three LIWC categories can be found in Text S1. To our knowledge the first two categories have not been validated before.

**Table 3:** Description of LIWC measures (as per http://www.liwc.net).

	Dictionary size	Examples
Anger	91	worried, fearful, nervous
Anxiety	84	hate, kill, annoyed
Sadness	101	crying, grief, sad
Tentative	155	maybe, perhaps, guess
Certainty	83	always, never
Fillers	9	blah, you know
Past	155	went, ran, had
Present	169	is, does, hear
Future	48	will, gonna
Social words	455	mate, talk, child

#### SentiStrength

This is a very recent word-counting tool [54], and is considered the state-of-the-art lexicon method for sentiment detection in short web texts [56]. Based on the 2007 revised version of LIWC, SentiStrength also accounts for modes of textual expression specific to the online environment, e.g. emoticons and abbreviations.

SentiStrength also provides a positive and a negative score for emotional valence. It is an adapted version of LIWC, much more appropriate for social media analysis. We analyze sentiment at comment level and then average across them for each editor. The SentiStrength emotion score is calculated at the sentence level, and then summarized at the comment level. At the sentence level, SentiStrength detects the number of positive and negative words. At the comment level, SentiStrength offers two different ways to compute the summarization:

- based on the mode of the sentence scores (e.g., if the most frequently encountered positive score for the sentences in a comment is 2, then the comment receives a score of 2)
- based on the strongest positive and negative emotion expressed in a comment (e.g., if the maximum positive score for the sentences in a comment is 2, then the comment receives a score of 2).

We chose to discard the SentiStrength scores based on the mode of the emotional value of the sentences, given that a problematic situation arises when the distribution of scores is multi-modal. Moreover, most of academic research conducted with SentiStrength focuses on the results based on the maximum valence words.

To increase the results comparability of the different lexicons, we also ran an ANEW-weighted version of SentiStrength (SentiStrength scores weighted by the number of ANEW words found in the comment from which the score is derived, so that each sentence has the same importance with the two lexicons). In the paper we report the non-weighted scores, since the same results hold for the weighted version of the scores. Consequently, the Results section below contains only this form of score computation, under the subheading “SentiStrength”.

### ANEW

For comparison purposes with [20], we present below a short description of the lexicon. The Affective Norms for English Words (ANEW) is a list of words with emotional scores for valence collected from human raters on a scale from 1 to 9 [55]. As opposed to SentiStrength and LIWC, most words in the ANEW list are not feeling-related words, i.e., they do not directly reference emotion (such as *happy* or *sad*); instead, they cover the entire spectrum of valence (including neutrality), and describe concepts that trigger associated emotions.

#### Example messages

In the following we use the example comments from [20] to illustrate the differences between the three lexicons. The marked comments in Table 4 can be understood as follows: bold-faced words have been identified in the SentiStrength library, words in italics are part of the LIWC dictionaries, while underlined words have been found in the ANEW lexicon. Should a word be both bold and in italic (e.g., challenge), this signifies that it has been found both in the SentiStrength and LIWC lexicons, and so on.

**Table 4:** Example messages with their corresponding LIWC *Pos*itive and *Neg*ative scores; SentiStrength Positive (P+) and Negative scores (N-); and ANEW Valence scores.

	LIWC		SentiStrength		ANEW
	Pos	Neg	P+	N-	V
Sounds like a ***good*** ** ***challenge*** - to be proven or disproven. I'm ***happy*** if it can be shown to go further using closed cubic polynomial solutions. The ***nice*** thing about these are that they are ***pretty*** * easy* to test numerically –in “Exact trigonometric constants”	15	0	3	-2	7.4
Seems you have not yet seen female ***lover*** after having sex who do not wish to have sex with the same ***lover*** any more **:)** Once you've seen it, you understand very *well* what ***war*** ** of Venus means compared to ** ***war*** ** of Mars.**–in “House (astrology)”	6.8	4.5	4	-3	5.5
What about the whirlie hazing, the alcohol ***abuse***, the ***emotional*** **poverty**, the **suicide** in 1995/6, the biotech plans which were stopped by pitzer ***protests*** –in “Harvey Mudd College”	4	8	1	-4	1.6

Words are written in *italic* if they contribute to the LIWC scores, in **bold** in the case of SentiStrength and words of the ANEW dictionaries are underlined.

We observe a high degree of overlap between the three lexicons, in terms of recognized words. However, the lexicons differ in terms of the scores assigned to the words. For example, the second comment includes the words “sex”, “lover” and “war”, and is therefore highly polarized/ambivalent regarding sentiment. The comment is composed of 44 words, out of which 3 are marked as positive in the LIWC lexicon (“well” and two times “lover”) and 2 as negative (two times “war”) which translates into a positive LIWC Score of 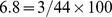 and a negative score of 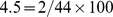. When using SentiStrength we detect the words with the strongest positive and negative emotions. For the example SentiStrength correctly indicates that the sentence contains both high positive emotion (+4), caused by the word “lover”, and negative emotion (-3), caused by the word “war”; as does LIWC. For comparison we also indicate the the valence level in ANEW, which loses information through averaging (5.5) over the disperse emotional scores associated to the words (indicated via underlining) of the ANEW lexicon. This illustrates, among others, the lexicons' similarities and complementarities.

### Network analysis

To analyze the network characteristics of emotions in Wikipedia, we computed scores for “emotional congruence” (similarity level of message-reply pairs) and “emotional homophily” (similarity of editors' emotional profiles). While emotional congruence is derived from the network of exchanged messages, homophily is based on a network of editors constructed by the rule that the editors included in the network have exchanged at least one reply. Emotional congruence is calculated as the average of the difference between the score of each comment and the score of the comment to which it replies; for this analysis all comments were considered, not just those coming from our set of highly active discussants. Meanwhile, to measure homophily, after aggregating comments at editor-level, assortativity is computed in the network using the *shuffle* test, as explained in detail below.

### Statistical tests

#### Nonparametric Tests

We first assessed the normality of the distributions of our variables of interest using Kolmogorov-Smirnov tests of normality. Since most variables are not normally distributed despite the large sample size, we examine the differences between Wikipedia editor groups by computing two-tailed Mann-Whitney U-tests which have a greater efficiency than t-tests on non-normal distributions. To increase the readability of tables, we highlight differences that are significant with 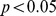 by showing the corresponding p-value in bold, and underlining the larger of the two population averages. For the cases (marked with an asterisk *) in which the averages were not informative, we include the mean ranks and underline the larger value. The sample size for the tests differs depending on whether the analysis was conducted on article or personal talk pages (considering that not all editors have a personal page and not all editors that have a personal page write or receive messages). However only 445 (6%) of the editors in our sample do not have a personal talk page. This percentage is even lower for administrators or for editors whose gender we were able to identify and can be neglected as possible explanation for the differences we observe in our analyses. The samle size also differs whether the analysis was conducted with LIWC or SentiStrength (for a number of editors SentiStrength could not classify the messages as strongly positive or negative). An overview of the test samples can be found in Table S1.

We also use one-tailed sign-tests (against the hypothesis that the differences are either larger or smaller than 0) to assess the significance of the differences observed when measuring emotional congruence.

#### Assortativity with shuffle test

To measure assortativity in the network according to a certain variable of the nodes, we compute the correlation (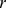) between the value of this variable for each pair of connected nodes, and we then perform a *shuffle test* [57]. This test is based on the comparison of the network with a number (in our case 100) of randomized equivalents, i.e. networks in which nodes have the same characteristics and the same number of connections (degree) as in the original, but links between nodes are randomly shuffled. Significance of the observed pattern is thus measured via the z-score, 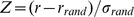, where 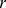 is the correlation coefficient for the desired variable in the original network, while 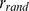 and 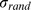 are, respectively, the average and the standard deviation of the same correlation measured in the randomized networks.

## Results and Discussion

We compare editors across two dimensions: status (Admins versus Non-admins) and gender (Male versus Female), and two lexicons: LIWC and SentiStrength. While we do not repeat the ANEW results presented in [20], we frequently address them as a means of cross-validation. We compare across both article talk pages and personal (“user talk”) pages. We conducted a correlation analysis between the per editor metrics of their comments in article and personal talk pages and found low correlations (at maximum of 0.35). This suggests that the editors' speech differs in the two spaces and justifies their separate analysis. The section ends with a network-level similarity assessment of the emotions expressed by editors who interact with one another.

### Emotions and Status

We first investigate differences in emotion and language according to status in Wikipedia. The LIWC results for the article talk pages shown in Table 5 suggest that admins express, on average, more positive emotion than regular editors. The result is significant also for the personal talk pages (see Table 6), suggesting that the positive attitude extends to the more private sphere of personal pages. Moreover, both in article and personal pages administrators refrain from using negative emotion, and the comments they receive also contain less negative emotion.

**Table 5:** Emotions and Status: Administrators promote a generally neutral tone on article talk pages.

(Article Talk)	Regular	Admin	Mann-Whitney U-Test	p-value
**LIWC**				
Positive	2.369	2.409	−4.308	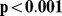
Negative	1.368	1.120	−18.578	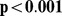
Affect	3.784	3.661	−8.466	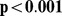
Anxiety	0.180	0.166	−5.834	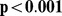
Anger	0.554	0.446	−19.217	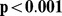
Sadness	0.175	0.166	−4.450	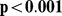
**SentiStrength**				
Positive	1.805	1.774	−14.603	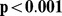
Negative	−2.005	−1.912	−23.046	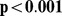

Regular editors express more negative emotion, and are more emotional.

Numbers under the editor class names correspond to the average values over all editors in a given class (sample size 12 231: 8 005 regular editors, 4 226 administrators). When the difference is statistically significant (p-value in bold) the larger absolute value is underlined.

**Table 6:** Emotions and Status: Admins are more emotional in personal talk pages compared to the article talk pages - they express more positive emotion compared to regular editors, but also more anxiety and sadness.

	Messages written				Messages received			
(Personal Pages)	Regular	Admin	U-Test	p-value	Regular	Admin	U-Test	p-value
**LIWC**								
Sample Size	6 644	4 086			6 636	4 086		
Positive	1.975	1.921	−3.553	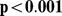 *	1.876	1.961	−8.751	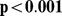
	(5282)	(5500)						
Negative	0.817	0.712	−5.211	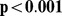	0.574	0.548	−7.229	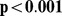
Affect	2.850	2.678	−4.299	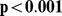	2.500	2.547	−0.587	p = 0.557
Anxiety	0.096	0.095	−13.476	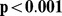 *	0.067	0.070	−5.977	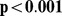
	(5060)	(5860)						
Anger	0.331	0.252	−3.408	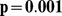	0.239	0.197	−10.312	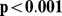
Sadness	0.096	0.094	−9.874	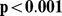 *	0.074	0.078	−7.076	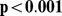
	(5141)	(5729)						
**SentiStrength**								
Sample Size	6 198	3 982			6 198	3 957		
Positive	1.98	1.96	−2.415	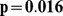	1.96	1.95	−2.951	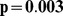
Negative	−2.03	−1.91	−13.618	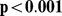	−1.85	−1.78	−16.618	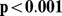

Numbers under the editor class names correspond to the average values over all editors in a given class. When the difference is statistically significant (p-value in bold) the larger absolute value is underlined. Cases where the averages are not informative are marked with an asterisk * and include the mean ranks Mann-Whitney U-test below the averages in parentheses.

These findings relate well to De Choudhury et al.'s [58] results on affect at the workplace. Their study focuses on messages exchanged via an internal microblogging tool at a large company, and shows that managers use more positive and less negative language when conversing with regular employees. In our case, we provide similar evidence for this phenomenon in a self-organized online collaborative environment.

Finally, the SentiStrength analysis adds interesting nuances to the results. It suggests that in article and personal pages (Tables 5 and 6) non-admins have a “higher pitch” when expressing both positive and negative emotion. Therefore, when they use positive and negative emotion words, they use stronger ones than the admins. Moreover, our analyses show (see right columns of Table 6) that regular editors receive stronger positive and negative words on their personal pages compared to admins, indicating that the “higher pitch” may be reciprocated.

When comparing admins and non-admins in terms of the discrete emotions they express, a distinctive picture emerges. The LIWC analysis suggests that regular editors are, on average, more emotional than admins. In article talk pages they express more affect, and in particular more anxiety, anger and sadness compared to admins (Table 5). The lower emotionality in administrators' communications is corroborated by the findings based on ANEW, reported in [20], that admins use less emotional content, as suggested by the lower arousal. Administrators' neutrality along with the increased referencing of Wikipedia policies [20] may be an expression of administrators' higher task-orientation, i.e., focus on setting goals and accomplishing tasks.

The results with regard to discrete negative emotions are mixed for the personal talk pages (Table 6). Administrators refrain from expressing negative emotion in general, but this does not hold for two particular emotions: anxiety and sadness. Similarly, the comments admins receive also contain more anxiety and sadness. This finding suggests that administrators are more relaxed with the expression of emotion within the “private” spaces of personal talk pages, while being impersonal in article talk pages.

The fact that administrators receive more anxiety and sadness (but not anger) on their personal pages may suggest a possible “ingratiation” strategy [5]. Anxiety and sadness are signals of personal vulnerability [59], [60], and coupled with the lack of anger, may indicate submissiveness towards the admins. Therefore, the fact that admins also receive more positive emotion and overall less negative emotion, could be interpreted as part of an ingratiation strategy [5], i.e., regular editors may wish to come across as attractive or likeable to higher status Wikipedians. A similar strategy has been documented by Danescu-Niculescu-Mizil et al. [41], who report that regular editors of Wikipedia change their linguistic style to match that of administrators. We provide evidence that, apart from language, emotion can also be used to characterize power imbalance in a collaborative environment.

To conclude, admins are more neutral in communication than regular editors in the public space of article talk pages. This is in line with previous literature [27], [42], which finds a tendency for increasing emotion neutrality and formalization as editors gain more experience in the community. When admins express emotion, they do so in a moderately positive manner. Our findings support the ones with ANEW reported in [20], and go in accordance with the idea that administrators generally have a positive tone and wish to embody the Wikipedian spirit of collaboration characterized by “good faith” [61]. Meanwhile, non-admins are more effusive in their emotional expression.

### Dialogue and Status

With the goal of bringing additional understanding to the differences in emotionality between admins and non-admins, we took advantage of the numerous text analyses that LIWC provides and compared Wikipedia editors across several dimensions of linguistic expression reported in Table 7 and 8, to understand how the differences can be understood in light of the status relations existent in the community.

**Table 7:** Dialogue and Status: Administrators are more impersonal in article talk pages. Regular editors are more concerned with others.

(Article Talk)	Regular	Admin	Mann-Whitney U-test	p-value
**Relationship-orientation**				
Personal pronouns	5.135	4.815	−13.561	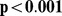
Use of “I”	2.456	2.429	−1.733	p = 0.083
Use of “You”	1.043	0.892	−12.573	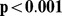
Use of “Shehe”	0.609	0.526	−8.657	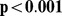
Social words	6.320	5.810	−19.013	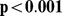
**Certainty**				
Certainty	1.426	1.317	−16.824	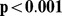
Tentativeness	3.199	3.169	−2.210	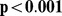
Filler words	0.168	0.155	−6.687	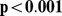
**Temporal Orientation**				
Past	2.376	2.305	−5.696	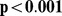
Present	8.011	7.841	−8.060	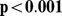
Future	1.114	1.166	−9.887	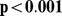

Numbers under the editor class names correspond to the average values over all editors in a given class (sample size 12 231: 8 005 regular editors, 4 226 administrators). When the difference is statistically significant (p-value in bold) the larger absolute value is underlined.

**Table 8:** Dialogue and Status: Regular editors send messages in a socio-emotional speech style. Administrators are more socially detached.

	Messages written				Messages received			
(Personal Pages)	Regular	Admin	U-test	p-value	Regular	Admin	U-test	p-value
Sample Size	6 644	4 086			6 636	4 086		
**Relationship-orientation**								
Personal pronouns	6.536	6.194	−7.256	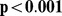	5.345	5.506	−0.459	p = 0.646
Use of “I”	3.758	3.519	−4.840	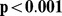 *	2.176	2.512	−14.047	p = 0.083
	(5478)	(5180)						
Use of “You”	1.772	1.704	−2.246	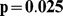 *	2.335	2.139	−18.321	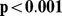
	(5312)	(5451)						
Use of ”Shehe”	0.407	0.350	−4.284	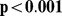 *	0.264	0.291	−7.939	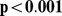
	(5266)	(5526)						
Social words	5.151	4.847	−6.831	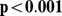	5.489	5.187	−19.941	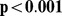
**Certainty**								
Certainty	0.776	0.724	−2.639	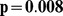	0.592	0.612	−0.095	p = 0.924
Tentativeness	2.358	2.452	−6.922	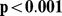	2.448	2.398	−9.859	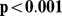
Filler words	0.004	0.001	−3.641	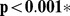	0.001	0.001	−13.288	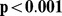 *
	(5344)	(5399)			(5233)	(5569)		
**Temporal Orientation**								
Past	2.258	2.235	−1.419	p = 0.156	1.730	1.884	−11.967	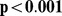
Present	6.496	6.381	−3.266	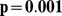	5.793	5.797	−9.043	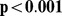 *
					(5573)	(5017)		
Future	0.891	0.928	−8.798	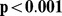	0.911	0.891	−7.549	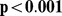

Numbers under the editor class names correspond to the average values over all editors in a given class. When the difference is statistically significant (p-value in bold) the larger absolute value is underlined. Cases where the averages are not informative are marked with an asterisk * and include the mean ranks Mann-Whitney U-test below the averages in parentheses.

#### Relationship-orientation

Compared to the rather impersonal comments of admins, regular editors connect more to other people. In article discussions and (to a somewhat lesser degree) in personal walls, they make more other-references (more personal pronouns) and use more words related to the social domain, such as mentions of friends and family.

Together with the poignant emotional expression of regular editors, this is a traditional marker for a socio-emotional speech style – non-admins think about the people in the conversation and try to establish a close relationship between themselves and the audience, whereas administrators are more socially detached. Our findings are in contrast with the results of Zhu et al. [62] who find that both admins and regular editors engage predominantly in task-oriented communication, i.e. communication focused on goal setting and task accomplishment [10]. This may be due to the very different conceptualization of task-oriented versus relationship-oriented language in their paper.

Further research is needed to determine whether the preoccupation with others is a consequence of the need to obtain the approval of higher status Wikipedians, and whether this is associated with higher levels of ingratiation and overaccomodation, as per Dino, Reysen and Branscombe [5]. In Wikipedia the community life is important and the prospect of being evaluated by peers (e.g., in order to attain administrator status) may motivate editors to maintain good relationships with higher authority community members.

#### Certainty

In line with previous literature on the speech insecurity of the powerless [32], we find that non-admins are less confident in article talk pages. They are generally more preoccupied with the topic of certainty, and use more tentative words (e.g., perhaps, maybe), and more filler words (e.g., errr, hmmm). We find little evidence of speech insecurity in personal pages: non-admins use fewer tentative and filler words compared to admins. This suggests that regular editors communicate more confidently within their personal space.

#### Temporal Orientation

Admins are more focused on the future, while regular editors seem more concerned with the present (and in article talk pages, also with the past). This may be related to a more pragmatic attitude of administrators, especially interested in the actions to be undertaken and “getting things done”. Considering also the more neutral tone of administrators, the interest in the future may be a reflection of their higher task-orientation, characterized by directive and instrumental speech.

#### Discussion

Interestingly, regular editors are more insecure only during discussion on article talk pages, indicative of the power imbalance within the public space of article discussion; for the personal pages this result does not hold, i.e. editors use more self-assured language within their personal space confirming the results from a question-answering system where experienced contributors were found to give more neutral answers [27], our detailed analyses of language suggests in wide agreement that admins tend, indeed, towards neutrality – they “rule with reason”, are more formal and impersonal in their discussion of Wikipedia articles compared to regular editors, while generally keeping the tone positive. Future research would be needed to assess to what extent the positive tone of administrators could in fact be due to sarcasm [63], [64].

In contrast, regular editors are characterized by a socio-emotional, people-oriented speech style, possibly as a means of ingratiation. Especially on article talk pages they are emotional and personal, reference others more, express more negative emotions, and are more effusive with all basic emotions by using stronger emotions words. Regular editors are also more concerned with the past and the present, while admins show a more pragmatic interest in the future.

We suggest that a potential explanation for the observed differences in emotion and language use could be administrators' tendency towards task-orientation compared to regular editors' leaning towards relationship-orientation within the Wikipedia community. This is an important distinction, considering that Zhu, Kraut and Kittur [24] investigated leadership behaviors across all levels of hierarchy in Wikipedia and found that task-oriented leadership had a mixed effect on contributions (with the transactional component being the most effective and the aversive one being the most detrimental), while relationship-oriented leadership had the strongest positive effect on contributions. Future research should determine how the communication style of higher status editors influences growth and, possibly, stagnation in online collaboration.

Finally, in an organizational setting, De Choudhury et al. [58] interpreted the positive tone of higher-status employees (managers and executives) as a manifestation of transformational leadership, a leadership style characterized by the commitment to inspire, excite and maintain high motivation levels in workers, e.g. by communicating an uplifting vision for the future [65]. Our study finds that positive emotion is only one piece of the puzzle, and future research is needed to indicate whether positive emotion may in fact co-occur with task-oriented communication.

### Emotions and Gender

In the following, we extend our analyses and examine gender differences in terms of emotional expression and language, aiming to bring much needed insight to the gender gap issue in Wikipedia editorship and in ICT, in general. Previous research with ANEW suggests that women express higher valence than men in article talk pages [20]. In the following we focus on the results from the LIWC and SentiStrength analyses, which characterize written expression on more than just valence.

#### Positive emotion

The LIWC lexicon (Tables 9 and 10) suggests that women indeed express more positive emotion than men during discussion both in article and personal talk pages. The results obtained using SentiStrength follow a similar pattern – women display more high-pitched valence, i.e., they use words that have a stronger positivity than men. This is in line with previous literature, regarding both the online and offline context [37], [38], [44], [66]. However, the above found differences might disappear when taking into consideration the topics on which women and men choose to work on, since women prefer topics discussed in a markedly positive manner [20]. Indeed, research on gender language differences has only recently considered the topic of conversation. Laniado et al. [20] provide indication that there are no gender differences in emotional expression when controlling for topic, but further research in a natural setting is needed to shed light on this issue.

**Table 9:** Emotions and Gender: Female Wikipedia editors express more positive emotion than male editors.

(Article Talk)	Men	Women	Mann-Whitney U-test	p-value
**LIWC**				
Positive	2.395	2.503	−3.064	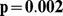
Negative	1.251	1.228	−0.192	p = 0.848
Affect	3.688	3.785	−1.928	p = 0.054
Anxiety	0.168	0.167	−0.740	p = 0.459
Anger	0.474	0.432	−1.094	p = 0.274
Sadness	0.168	0.182	−0.044	p = 0.965
**SentiStrength**				
Positive	1.776	1.800	−3.160	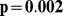
Negative	−1.929	−1.936	−0.539	p = 0.590

Expression of negative emotion is similar for men and women.

Numbers under the editor class names correspond to the average values over all editors in a given class (sample size 2 778: 2 613 men and 165 women). When the difference is statistically significant (p-value in bold) the larger absolute value is underlined.

**Table 10:** Emotions and Gender: Female editors express more positive emotion than males, while the expression of negative emotion is generally similar for men and women.

	Messages written				Messages received			
(Personal Pages)	Men	Women	U-test	p-value	Men	Women	U-test	p-value
**LIWC**								
Sample Size	2 446	160			2 445	160		
Positive*	1.896	2.433	−2.724	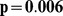 *	1.918	2.030	−3.644	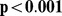
	(1293)	(1460)						
Negative	0.707	0.732	−0.262	p = 0.794	0.551	0.546	0.631	p = 0.821
Affect	2.647	3.207	−2.533	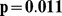	2.507	2.616	1.584	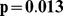
Anxiety	0.104	0.102	−1.218	p = 0.223	0.069	0.069	1.123	p = 0.160
Anger	0.246	0.263	−0.308	p = 0.758	0.201	0.184	0.794	p = 0.554
Sadness	0.092	0.092	−0.523	p = 0.601	0.079	0.085	1.262	p = 0.083
**SentiStrength**								
Sample Size	2 358	156			2 349	154		
Positive	1.96	2.01	−2.724	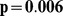 *	1.95	2.01	−5.757	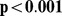
	(1247)	(1410)						
Negative	−1.93	−1.93	−0.087	p = 0.930	−1.79	−1.78	−0.913	p = 0.361

Numbers under the editor class names correspond to the average values over all editors in a given class. When the difference is statistically significant (p-value in bold) the larger absolute value is underlined. Cases where the averages are not informative are marked with an asterisk * and include the mean ranks Mann-Whitney U-test below the averages in parentheses.

The difference in positive emotion remains significant when comparing men and women at the administrator level with both LIWC and SentiStrength, thereby contradicting the results obtained with ANEW [20]. On the other hand, female administrators are similar to male administrators with respect to all other types of emotion in article pages (Table 11). For personal talk pages female admins appear more emotional than male admins for both sent and received messages, suggesting that women restrict their emotionality to a certain extent in the public space of article talk (Table 12).

**Table 11:** Emotions, Gender and Status: Wikipedia female administrators express more positive emotion than male administrators in article talk pages, but are similar in the expression of negative emotion.

(Article Talk)	Men	Women	Mann-Whitney U-test	p-value
**LIWC**				
Positive	2.419 (805)	2.502 (911)	−2.147	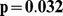 *
Negative	1.197	1.183	−0.464	p = 0.643
Affect	3.656	3.739	−1.334	p = 0.182
Anxiety	0.163	0.156	−1.519	p = 0.129
Anger	0.440	0.401	−0.985	p = 0.325
Sadness	0.163	0.185	−0.201	p = 0.841
**SentiStrength**				
Positive	1.766	1.778	−1.663	p = 0.096
Negative	−1.900	−1.915	−0.900	p = 0.368

Numbers under the editor class names correspond to the average values over all editors in a given class (sample size 1 623 administrators: 1 526 men, 97 women). When the difference is statistically significant (p-value in bold) the larger absolute value is underlined. Cases where the averages are not informative are marked with an asterisk * and include the mean ranks Mann-Whitney U-test next to the averages in parentheses.

**Table 12:** Emotions, Gender and Status: Wikipedia female administrators are more emotional than male administrators in personal talk pages.

	Messages written				Messages received			
(Personal Pages)	Men	Women	U-test	p-value	Men	Women	U-test	p-value
**LIWC**								
Sample Size	1 487	94			1 487	94		
Positive	1.921	2.045	−2.767	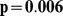 *	1.938	2.012	−2.482	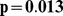 *
	(783)	(917)			(783)	(904)		
Negative	0.688	0.726	−0.426	p = 0.670	0.546	0.570	−1.888	p = 0.059
Affect	2.650	2.819	−2.225	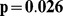 *	2.520	2.621	−2.841	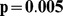 *
	(784)	(892)			(782)	(920)		
Anxiety	0.096	0.10	−2.094	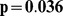 *	0.069	0.073	−2.005	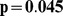 *
	(785)	(885)			(785)	(882)		
Anger	0.243	0.218	−0.277	p = 0.782	0.193	0.191	−1.013	p = 0.311
Sadness	0.089	0.102	−1.123	p = 0.262	0.079	783	916	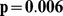
**SentiStrength**								
Sample Size	1 446	94			1 446	92		
Positive	1.95	2.00	−2.285	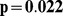 *	1.94	2.00	−5.219	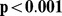
	(766)	(875)						
Negative	−1.91	−1.91	−0.230	p = 0.818	−1.78	−1.79	−1.155	p = 0.248

Numbers under the editor class names correspond to the average values over all editors in a given class. When the difference is statistically significant (p-value in bold) the larger absolute value is underlined. Cases where the averages are not informative are marked with an asterisk * and include the mean ranks Mann-Whitney U-test below the averages in parentheses.

It must be said, however, that male administrators are found to be more positive than regular male editors (Table 13) in the article talk pages, possibly indicating that they promote a positive working environment, pivotal to the good functioning of the Wikipedia community. On the other hand, male admins express less emotion compared to regular editors. In fact, they differ significantly from the regular counterparts on almost all basic and discrete emotion dimensions with respect to article discussions. We do not observe this result for the personal pages, suggesting that male administrators adhere to administrator role expectations only within the public space of article discussions.

**Table 13:** Emotions, Gender and Status: Wikipedia male regular editors differ significantly from male admins.

(Article Talk)	Regular	Admin	Mann-Whitney U-test	p-value
**Men**				
Sample Size	1 087	1 526		
LIWC Positive	2.362	2.419	−3.046	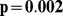
LIWC Negative	1.326	1.197	−6.415	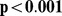
LIWC Affect	3.733	3.656	−1.844	p = 0.065
LIWC Anxiety	0.176	0.163	−2.852	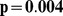
LIWC Anger	0.476	0.401	−6.184	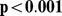
LIWC Sadness	0.176	0.163	−2.957	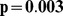
SentiStrength Positive	1.790	1.766	−5.052	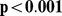
SentiStrength Negative	−1.970	−1.900	−8.319	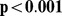
**Women**				
Sample Size	68	97		
LIWC Positive	2.503	2.502	−0.541	p = 0.588
LIWC Negative	1.293 (93)	1.183 (75)	−2.316	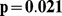 *
LIWC Affect	3.850	3.739	−0.952	p = 0.341
LIWC Anxiety	0.184 (91)	0.156 (76)	−1.996	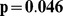 *
LIWC Anger	0.476	0.401	−1.848	p = 0.065
LIWC Sadness	0.179	0.185	−1.107	p = 0.268
SentiStrength Positive	1.832	1.778	−2.759	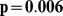
SentiStrength Negative	−1.969	−1.789	−1.472	p = 0.141

Women regular editors express more negative and stronger positive emotions compared to women admins.

Numbers under the editor class names correspond to the average values over all editors in a given class. When the difference is statistically significant (p-value in bold) the larger absolute value is underlined. Cases where the averages are not informative are marked with an asterisk * and include the mean ranks Mann-Whitney U-test next to the averages in parentheses.

Lastly, our analysis with LIWC shows that women non-admins express more negative emotions, anxiety and anger in particular, than women admins during conversation on article and personal pages. This comes in contrast with the results of [20] who find that women non-admins have similar emotional profiles to women admins. On the other hand, SentiStrength suggests a slightly different pattern for the article talk pages – women non-admins are more effusive in the expression of strong positive emotion, while being similar with regard to strong negative emotion expression, thereby confirming the findings of Laniado et al. [20] (Table 13).

#### Negative emotion

In contrast to the mixed results in previous literature [38], [46], [47], our analyses find no significant gender difference in negative emotional expression, which is the case for both article (Table 9) and personal talk pages (Table 10). This holds for both LIWC and SentiStrength in terms of overall negative emotion, as well as in particular for anxiety and sadness.

#### Anger

In contrast with previous literature [37], [38], which suggests that women express less anger, we do not observe this effect, neither for article nor for personal talk pages. Regarding male anger expression we observe that male admin comments in article talk pages contain less anger than those of male non-admins, which corroborates our previous results of administrator neutral tone. Moreover, male admins receive less anger in personal pages, supporting the ingratiation hypothesis we presented previously.

#### Discussion

All lexicons find unanimously that women express more positive emotion compared to men, which might in fact be owing to topic choice of male versus female editors, as observed in [20]. More novel is that male administrators are quite different from male normal editors in terms of emotional expression.

The fact that administrators (in particular, male administrators) are more neutral, more formal (they reference Wikipedia policies more often) and less concerned with other Wikipedia editors could indicate that they are more task-oriented. On the other hand, regular editors (females, in particular) are clearly more relationship-oriented than their higher-status counterparts.

### Dialogue and Gender

In the previous section we have compared the emotional expression of male and female editors in Wikipedia talk pages. Further on, we increase our understanding of gender differences by characterizing editor style along other dimensions of linguistic expression, as done for status.

#### Relationship-orientation

One of our most robust findings is that women and men differ in terms of the preoccupation with the social domain (see Tables 14 and 15), consistent with the observation that women are more interested in relating to others, i.e., in building and maintaining relationships. Women are more sociable compared to men, since they use more socially-related words, and make more self- and other-references, e.g., use of more personal pronouns. The personal pages of women are also found to be more social than those of men, and in particular here other-referencing is more common.

**Table 14:** Dialogue and Gender: Female editors use a relationship-oriented speech style.

(Article Talk)	Men	Women	Mann-Whitney U-test	p-value
**Relationship-orientation**				
Personal pronouns	4.964	5.420	−4.375	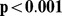
Use of “I”	2.488	2.764	−3.945	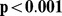
Use of “You”	0.936	0.957	−0.926	p = 0.355
Use of “Shehe” pronouns	0.541	0.713	−4.657	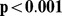
Social words	5.960	6.353	−3.487	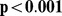
**Certainty**				
Certainty	1.346 (1397)	1.300 (1263)	−2.078	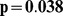 *
Tentativeness	3.150	3.215	−1.162	p = 0.245
Filler words	0.161	0.160	−0.137	p = 0.891
**Temporal Orientation**				
Past	2.325	2.543	−4.305	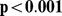
Present	7.897	8.180	−3.086	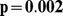
Future	1.168	1.147	−1.008	p = 0.314

Numbers under the editor class names correspond to the average values over all editors in a given class (total sample 2 778 editors: 2 613 men 165 women). When the difference is statistically significant (p-value in bold) the larger absolute value is underlined. Cases where the averages are not informative are marked with an asterisk * and include the mean ranks Mann-Whitney U-test next to the averages in parentheses.

**Table 15:** Dialogue and Gender: Female editors write and receive more relationship-oriented speech on personal talk pages.

	Messages written on own wall				Messages received			
(Personal Pages)	Men	Women	U-test	p-value	Men	Women	U-test	p-value
Sample Size	2 446	160			2 445	160		
**Relationship-orientation**								
Personal pronouns	6.225	6.874	−4.772	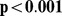	5.375	5.839	−4.587	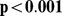
Use of ”I”	3.560	3.762	−3.232	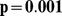 *	2.401	2.713	−3.610	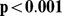
	(1291)	(1489)						
Use of ”You”	1.693	2.077	−3.070	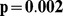	2.150	2.200	0.832	p = 0.493
Use of “Shehe”	0.345	0.438	−2.071	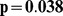 *	0.272	0.335	1.518	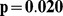
	(1295)	(1421)						
Social words	4.946	5.396	−3.499	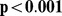	5.181	5.467	2.122	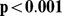
**Certainty**								
Certainty	0.717	0.697	−1.212	p = 0.226	0.596	0.618	0.704	p = 0.705
Tentativeness	2.390	2.243	−0.198	p = 0.843	2.361	2.335	0.605	p = 0.858
Filler words	0.001	0.001	−2.879	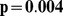 *	0.001	0.001	−2.185	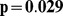 *
	(1300)	(1346)			(1299)	(1361)		
**Temporal Orientation**								
Past	2.223	2.119	−0.300	p = 0.764	1.823	1.898	0.938	p = 0.343
Present	6.392	6.509	−1.209	p = 0.227	5.679	5.754	0.839	p = 0.482
Future	0.922	0.890	−0.269	p = 0.788	0.878	0.868	0.962	p = 0.312

Numbers under the editor class names correspond to the average values over all editors in a given class. When the difference is statistically significant (p-value in bold) the larger absolute value is underlined. Cases where the averages are not informative are marked with an asterisk * and include the mean ranks Mann-Whitney U-test below the averages in parentheses.

We therefore add to the rather undisputed literature on women's' rapport interest [45], [66]; on the other hand, it is unexpected to find this result for the article talk pages, i.e., in a mixed-gender, non-personal topic context, contrary to the meta-analysis of Leaper and Ayres [45]. Even more revealing is that this finding holds across hierarchy levels in the community. Table 16 illustrates the strength of relationship-orientation across different editor groups. Specifically, we find that female regular editors are more people-focused than male regular editors, and the same holds for female administrators versus male administrators. Finally, female regular editors are more concerned with relationships than female admins, and similarly for male regular editors compared to male admins.

**Table 16:** Dialogue, Status and Gender: Male admins are the least relationship-oriented, female regular editors are the most relationship-focused.

	Relationship-Orientation				
(Article Talk)	Pers. Pron.	“I”	“You”	“Shehe”	Social
Male admins	4.868	2.481	0.893	0.509	5.791
Female admins	5.226	2.726	0.917	0.635	6.035
U-test	−2.973	−3.035	−0.745	−3.307	−1.911
p-value	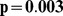	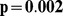	p = 0.456	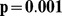	p = 0.056
Male regulars	5.099	2.498	0.996	0.585	6.198
Female regulars	5.697	2.817	1.014	0.823	6.808
U-test	−3.349	−2.545	−0.566	−3.343	−3.276
p-value	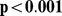	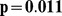	p = 0.571	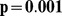	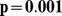
Female admins	5.226	2.726	0.917	0.635	6.035
Female regulars	5.697	2.817	1.101	0.823	6.008
U-test	−2.008	−0.358	−1.081	−1.573	−3.193
p-value	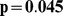	p = 0.721	p = 0.280	p = 0.116	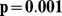
Male admins	4.868	2.481	0.893	0.509	5.791
Male regulars	5.099	2.498	0.996	0.585	6.198
U-test	−4.426	−0.695	−3.935	−3.146	−7.073
p-value	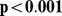	p = 0.487	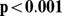	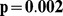	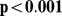

Numbers under the editor class names correspond to the average values over all editors in a given class (sample size 2 613 men: 1 087 regular editors and 1 526 administrators; and 165 women: 68 regular editors and 97 administrators). When the difference is statistically significant (p-value in bold) the larger absolute value is underlined.

All in all, regular editors are more relationship-focused than administrators, and women more than men. The least relationship-oriented are male administrators, while women regular editors are the most people-focused. Interestingly, this holds true only for the article talk pages. For the personal talk pages the only similar finding is that female admins are more concerned with other editors than male admins. We interpret the difference between article and personal pages in light of the public-private space dichotomy. While “at work” administrators adopt a more neutral, formal and instrumental speech in discussion of non-personal topics on article talk pages, but this difference attenuates in the more private sphere of personal pages.

#### Qualitative analysis

To support our assumption of a possible link between relationship-orientation and a personable approach towards others, we conducted a qualitative analysis of 100 comments from the article talk pages. We find evidence for the co-occurrence of the two interaction styles. Comments high in relationship-orientation are also high in “niceness”, reveal a genuine interest in understanding other editors' perspectives and generally indicate a collaborative attitude. The classification of the comments high in relationship-orientation according to content yields several types of comments: 1. inviting comments that explain the edit in a friendly tone, and call for further intervention and collaboration; 2. common perspective-building comments that are focused on understanding others and solving debates in a constructive manner; 3. appreciative comments that contain positive emotions and celebrate others' actions. This suggests that relationship-orientation may be conducive to successful collaboration and further research should shed light on this issue.

#### Self-referencing

An additional finding is that women self-reference more than men in the public space of article talk pages by using “I” and “I”-related pronouns. A possible explanation could be that women are more insecure, and express themselves using first person singular and uncertainty words (e.g. I guess, maybe), which is more compliant [67]. We find some evidence for this in our data, as we detect differences between men and women with regard to the use of certainty words in article talk pages and filler words in personal talk pages (see Tables 14 and 15).

Another potential explanation relates to the minority status that women have in Wikipedia. The psychological literature suggests that feeling distinctive within a group (e.g. being part of a minority) predicts self-focused attention [68], [69]. Therefore, if women Wikipedians are aware that they constitute a minority, this could trigger more self-focus.

Most likely, the use of the active first-person singular is a signal of heightened social sensitivity, expressing consciousness of the subjectivity of one's own point of view. By saying phrases such as “I think”, “I believe” and by expressing positive emotions, women may be engaging in relationship-oriented speech. Consequently, the use of “I” may be an indicator that they are considering perspectives other than their own – a personable approach to relating to others.

#### Certainty

We find minor differences between men and women with regard to the use of (un)certainty words (certainty words in article talk pages and filler words in personal talk pages - see Tables 14 and 15). This is an unexpected result, given that women's' lack of confidence in their ability to edit has been suggested as a possible cause for the gender gap in Wikipedia [51]. This does not appear to be the case for women highly active and involved in discussion (those in our sample, who pass the 100 comment-mark).

#### Temporal Orientation

Female editors are more concerned with the past and present. However, this finding holds only for article talk pages.

#### Discussion

One robust finding is that women are more interested in relating to others than men – they use more social words and reference themselves and others more. This is true at each hierarchy level in the community. Therefore, the use of more relationship-oriented language could help attract and retain more women to Wikipedia. Moreover, our qualitative analysis suggests that the higher relationship-orientation is connected to an open and constructive attitude towards collaboration, while Zhu, Kraut and Kittur [24] show that it increases contributions. Since women are more relationship-oriented, attracting them would create a positive circle and lead to a more inviting peer-production environment, ultimately triggering increased community morale and more contributions.

### Network characteristics of emotion and language

So far we have studied how emotions and language vary according to characteristics of the editors, namely status and gender. To do this, we have considered each comment separately, and we have then aggregated all comments of (or directed to) the same editor. In this section, on the contrary, we disregard individual editor characteristics, but we consider the relationships between comments and their replies to investigate how emotion and language relate to editor interactions. In particular, we investigate this at the comment level, studying how individual messages differ from the comments they reply to (*emotional congruence*) and at the editor level, studying how similar are the emotional profiles of editors who interact with one another (*emotional homophily*).

#### Emotional congruence

According to the ANEW results reported in [20], editors tend to reply with significantly (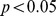) higher valence. The analyses with LIWC on the same dataset, including about 2.5 million comment-reply pairs, confirm this finding and contribute to its understanding. Here we use a non-parametric sign-test which does not assume any particular features of the tested distribution. In Table 17 we report the results of the sign-test on the differences between the metrics of comment and reply pairs. The table shows whether replies are more likely to contain larger or lower values of each metric; more specifically, the column “Overlay” indicates the difference between the number of cases when replies have a higher or a lower value of the corresponding metric; i.e, it indicates in which proportion it is more likely that replies have a higher value for that metric (or less likely, in case of a negative percentage).

**Table 17:** Emotional congruence: Surplus of positive vs. negative differences between metrics of replies and messages to which they reply.

(Article Talk)	Overlay	sign-statistic	p-value
**LIWC**			
Positive	4.3%	1194129	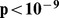
Negative	−1.3%	944750	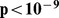
Affect	4%	1250178	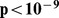
Anxiety	−3.6%	296025	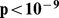
Anger	−3.6%	564067	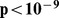
Sadness	−4.7%	285268	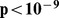
**Relationship-orientation**			
Personal pronouns	6%	1286441	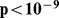
Use of “I”	1.4%	1124395	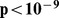
Use of “You”	10.5%	853112	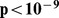
Use of “Shehe” pronouns	−5.3%	393313	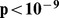
Social words	2.6%	1253751	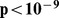
**Certainty**			
Certainty	−0.8%	1009401	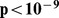
Tentativeness	−1.5%	1160532	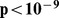
Filler words	−3.9%	280376	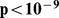
**Temporal Orientation**			
Past	−0.5%	1081248	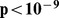
Present	1.6%	1254986	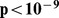
Future	−1.3%	954094	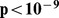
**SentiStrength**			
Positive	−0.9%	754158	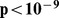
Negative	1.6%	781334	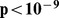

Percentages indicate how often a metric for a reply is larger than the corresponding metric for the replied comment. If the percentage is negative the difference between the two metrics is more often negative. Sample sizes: 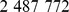 comment pairs for LIWC and 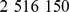 for SentiStrength.

We confirm [20] and find a surplus of replies that contain a higher percentage of positive words (4.3%) and consistently also an under-representation of replies that contain a higher percentage of negative words, although to a lesser extent (−1.3%). Both results are highly significant (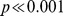).

Accordingly, replies tend to contain more emotional content (affect), but less anger, sadness and anxiety than the messages they reply to, confirming a general trend to keep a positive tone in discussions. Moreover, it is more likely that replies contain more first and second personal pronouns, especially “You” (10.5%), and more socially-oriented words (See Table 17). Together with the expression of positive emotion, these are all markers of a socio-emotional communication style which appears to characterize editors when replying.

Interestingly, SentiStrength results for congruence contrast with LIWC and ANEW results, and suggest that while more positive on average, replies tend to contain slightly less “high-pitched” positive emotions, and more high-pitched negative emotions.

#### Emotional homophily

By investigating homophily we can determine whether the editors that interact (exchange at least one message on article talk pages) have similar emotional and conversational profiles. According to the concept of *assortativity* [70], assortative networks are characterized by a preference to interact with similar others; meanwhile, in disassortative networks dissimilar people are more connected. We find two types of assortativity:

#### Assortativity according to emotions

Confirming the results shown for the ANEW variables in [20], the reply network is assortative for all emotion variables, with values of 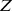 always greater than 2 (Table 18). Therefore, editors are more likely to interact with others having a similar emotional style. This is a confirmation of emotional homophily in the community, also for discrete emotions measured by the LIWC lexicon such as anger, anxiety or sadness.

**Table 18:** Emotional assortativity: large positive Z-scores indicate homophily in the “reply” network, according to emotions in the messages written by each editor.

(Article Talk)	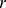	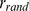	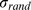	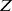
**LIWC**				
Positive	0.0797	−0.0001	0.0014	**57.0**
Negative	0.1900	0.0001	0.0014	**139.5**
Affect	0.1202	−0.0001	0.0016	**74.5**
Anxiety	0.0909	−0.0002	0.0013	**65.4**
Anger	0.2291	−0.0002	0.0014	**163.1**
Sadness	0.0891	0.0001	0.0016	**55.1**
**SentiStrength**				
Positive	0.1645	−0.0003	0.0014	**117.0**
Negative	0.3191	−0.0002	0.0014	**226.1**

All scores are statistically significant (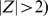.

**Figure 1:**
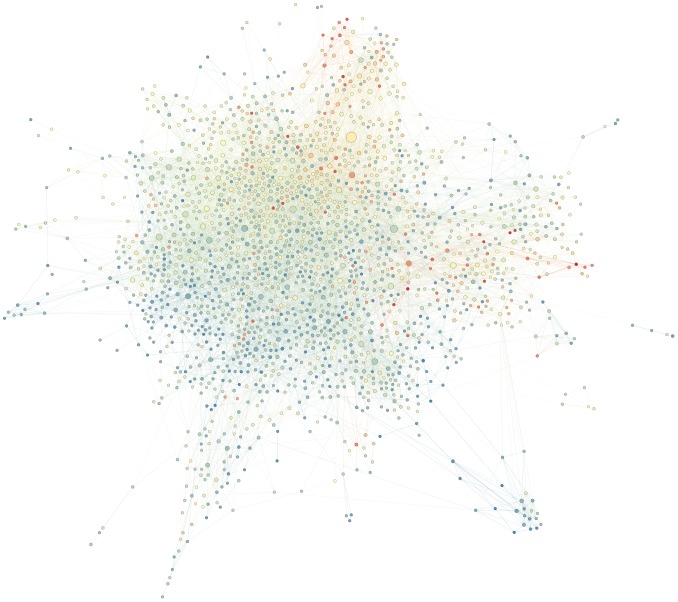
Assortativity in the reply network according to the expression of anger. The color of each node depends on the proportion of words expressing anger in the comments written by the corresponding editor, from blue (low) to red (high). Two editors are connected if they exchanged at least 10 replies in article talk pages. Node size is proportional to the number of connections.

Figure 1 illustrates the finding above for assortativity in terms of anger expressed in the messages. It depicts the network of replies, where editors are connected if they exchanged at least ten messages. Node colors range from blue for editors low in anger expression, to red for editors who use a high proportion of words expressing anger. One can visually observe that similar editors tend to “stick together”, i.e., connect more than dissimilar editors.

#### Assortativity according to other speech-related LIWC variables

Interestingly, as shown in Table 19, the reply network is largely assortative also with respect to all other language use variables considered in this study, indicating homophily also in terms of relationship-orientation, certainty and temporal orientation. Therefore, editors with similar emotional and conversational profiles tend to connect more in Wikipedia.

**Table 19:** Language assortativity: large positive Z-scores indicate homophily in the “reply” network, according to the communication style of the messages written by each editor.

(Article Talk)	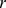	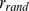	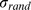	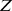
**Relationship-orientation**				
Personal pronouns	0.0998	−0.00042	0.0015	**66.4**
Use of “I”	0.0404	0.00001	0.0015	**26.2**
Use of “you”	0.1131	−0.00006	0.0015	**74.0**
Use of “Shehe”	0.1806	−0.00028	0.0015	**122.7**
Social words	0.1947	−0.00021	0.0014	**134.5**
**Certainty**				
Certainty	0.0789	0.00005	0.0015	**53.3**
Filler	0.0797	−0.00017	0.0014	**56.5**
Tentat	0.0758	−0.00054	0.0013	**59.4**
**Temporal Orientation**				
Past	0.1252	−0.00046	0.0015	**84.4**
Present	0.0922	−0.00044	0.0012	**80.5**
Future	0.0526	−0.00013	0.0014	**36.5**

All scores are statistically significant (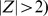.

While we do not report the results here, our analysis of the communication network for personal talk pages presents very similar patterns – the network is clearly assortative for all variables. In this analysis we considered the network of messages written by editors on one another's talk page, but still characterizing each editor with her style in article discussions. In this way we looked at personal communications in a network that is not based on the comments on which the scores are computed, so we could expect a different outcome. Instead, the results confirm assortativity for all variables, suggesting that editors who express emotion and communicate similarly on article talk pages also interact more with one another on personal pages.

#### Discussion

We find conclusive evidence for emotional homophily, a relatively widespread phenomenon in community sites [12], [13], [27]: editors tend to interact preferentially with others having a similar style, and expressing similar emotions. As noted in previous literature [13], there are two main explanations for this phenomenon: the first is that similar editors are more likely to interact with one another because they have similar interests, and the second is emotional contagion. It is of course difficult to separate these two phenomena, which are probably both present. However, our finding that editors having a similar style in article talk pages also communicate more with one another in personal spaces suggests that there is something more than just emotional contagion at the micro-level, and that indeed *birds of a feather flock together*. Interestingly, none of the observed emotional and linguistic features of speech makes an exception to this rule.

At the message-reply level, the results seem to confirm the finding of [20] of a general trend to exhibit a positive attitude when replying to other editors, in line with the community guidelines and netiquette (for example [71]). In fact, editors overall tend to reply with more positive emotion, more relationship-oriented language and more affect, but less anger, which can be seen as signs of an increased attention towards the others when replying. However, replies tend to contain more high-pitched negative words, which suggests a more complex picture of interactions at a micro-level; a more fine grained analysis, based on qualitative observation, could shed further light on this aspect, while a possible effect of negative replies on editor participation could be investigated by studying editor activity over time.

## Conclusions

Our results provide insights for the understanding of community evolution and engagement, and have implications for communities facing membership stagnation, similar to Wikipedia.

We find that higher-status editors promote a neutral, impersonal and more formal conversation tone in Wikipedia. They “rule with reason”, and maintain a mildly positive tone – a crucial aspect to the good functioning of the collaborative project. Nevertheless, it is not clear whether administrator neutrality fulfills the needs of the community in the long run. Peer-production communities are settings of voluntary contribution, and emotions play an important role in group dynamics, requiring expression. Relationship-oriented communication has been found to increase contributions [24] and, interestingly, regular editors use this linguistic style more than administrators. Consequently, the tone of group moderators, and more generally the interaction spaces of such communities should be adapted to facilitate both positive exchanges and the venting out of negative emotion in a constructive manner.

For this aim, the role of female editors is paramount. Indeed our analyses (both automatic and the brief manual analysis of content) provide strong evidence that female editors engage in relationship-oriented speech that is conducive to a positive working environment. Interestingly, this result holds also for female administrators, who diverge significantly from male administrators by being more relationship-oriented. By increasing the diversity of leadership styles and by promoting an atmosphere of openness and concern for others, women leaders play a pivotal role in such online spaces.

These results have implications also for the gender gap issue. Together with the finding of [20] that women tend to interact preferentially with other women, our results suggest that being able to involve more women and to give them more space in the community would also result in a virtuous cycle of female participation, through the creation of a communication environment where they feel more comfortable.

Furthermore, we identify a special group of Wikipedian women who look confident in their own abilities. Previous research suggested lack of self-confidence as a possible reason for the gender gap in Wikipedia [51]. This is not the case for women who are active discussants in the community, and who exhibit the same level of confidence as men, irrespective of their status in Wikipedia.

Beyond gender, our results indicate that the discussion network is highly assortative also with respect to emotion and communication style. Editors communicate more with others having a similar style, both in terms of emotional and communication profiles. This suggests that clusters of different emotions and linguistic styles can be identified and managed in collaborative communities.

Finally, we find a relation between hierarchy and emotional and linguistic patterns. Regular editors use more insecure expressions than administrators on article talk pages, but not on personal pages. This suggests that there is a perceived sense of hierarchy in Wikipedia (and possibly intensified by administrators' formal tone in communication). Further research should indicate to what extent and under which conditions a perceived hierarchy is beneficial to the long-term well-being of a collaborative community.

To conclude, this paper increases our understanding of peer-production processes, in terms of the emotional expressions and responses of contributors. We have provided data and insights about the emotional and conversational dimension of Wikipedia, and how emotions and language use are related to the profiles of editors and to their interactions.

Further lines of research include conducting a similar multi-metrics study within other communities, over a long time period, and considering also less active and anonymous editors in Wikipedia. Although we already relied on a combination of quantitative and qualitative approaches (including crowdsourcing to assess the validity of automatic techniques), a richer usage of human annotations and one that accounts for non-textual emotion (e.g., emoticons, “barn stars”, and virtual gifts) would certainly help to get a deeper and more fine-grained understanding of the results, as well as to provide valuable input to increase the performance of automatic methods. For example, it would be important to be able to detect in which contexts messages with high-pitched positive emotions are likely to be just sarcastic.

We would also like to incorporate a time dimension to study the effects of emotions. For instance, related to Wikipedia's decline in membership levels, it would be interesting to test if the editors that eventually abandon the community received more negative messages than the editors that stay and get more engaged. It would be also useful to analyze the vocabulary of the community, similar to [72], to investigate the relationship between the editor involvement and the adhesion to the community lexicon. Moreover, the emotion and communication metrics (e.g., positive emotion or socio-emotional style) could be linked to performance metrics of the collaborative effort, such as the quality of Wikipedia articles.

## Supporting Information

Text S1 **Lexicon validation.** A brief review of previous validation research of emotional lexicons and a description of our own efforts in this direction.
(PDF)

Table S1 Sample sizes for the Mann-Whitney tests for article and personal talk pages.
(PDF)
