# Supplementary material for: Emotions under Discussion: Gender, Status and Communication in Online Collaboration
Source: PLoS One. 2014 Aug 20;9(8):e104880. doi: 10.1371/journal.pone.0104880 (PMC4139304; doi:10.1371/journal.pone.0104880)
Supplement: Text S1 — Lexicon validation. A brief review of previous validation research of emotional lexicons and a description of our own efforts in this direction. (PDF) [file pone.0104880.s001.pdf]

SUPPORTING INFORMATION FOR: Emotions under Discussion: Gender, Status and Communication in Online Collaboration

Daniela Iosub[1], David Laniado[2], Carlos Castillo[3], Mayo Fuster Morell[4], Andreas Kaltenbrunner[2,*]
1 Cologne Graduate School, University of Cologne, Germany
2 Social Media Research Group, Barcelona Media Foundation, Barcelona, Spain
3 Qatar Computing Research Institute, Doha, Qatar
4 Berkman Center, Cambridge, MA, USA
∗ E-mail: Corresponding author andreas.kaltenbrunner@barcelonamedia.org

Text S1: Lexicon validation

Before making use of the lexicons, we checked previous literature for indication of their validity and, in the case of no available information for several LIWC categories, we proceeded to analyze the validity ourselves. Consequently, the following subsection presents a brief review of previous validation research, as well as our own efforts.

In the case of SentiStrength, we are aware of only one study investigating the performance of the lexicon. According to Thelwall, Buckley, Paltoglou, Cai and Kappas [1], SentiStrength detects sentiment relatively well – it is able to predict positive emotion with 60.6% accuracy and negative emotion with 72.8% accuracy, both based upon strength scales of 1-5.

The performance of the LIWC lexicon for the basic emotion dimensions, such as positive and negative valence, has been analyzed by Bantum and Owen [2]. They analyzed the sensitivity and the specificity of positive and negative emotion. Sensitivity was defined as "true positive" rate, i.e. the probability that a word that is actually representative of a (positive or negative) emotion is recognized by LIWC as being characteristic for that emotion. Specificity as the "true negative" rate. i.e. the probability that a word that that is not representative of an emotion would not be recognized by LIWC as characteristic for that emotion. Sensitivity and specificity values for positive emotion were 0.89 and 0.97, respectively, while for negative emotion they were 0.78 and 0.99.

On the other hand, the LIWC lexicon allows us to identify language differences that go beyond emotion expression, and may even help clarify the differences in emotional profiles among editor groups. However, the validity of the LIWC instrument has not been shown for all language categories assessed by the instrument. Indeed, there is very little independent work regarding the psychometric properties of LIWC, especially regarding its accuracy for categories other than emotional expression.

In our validation efforts we were interested particularly in the accuracy of LIWC categories concerning *relationship-orientation* and *certainty*, from which we derived insight into the linguistic and emotional differences between Wikipedia editors. In our study we define relationship-orientation as the preoccupation with the social domain, such as concern for and motivation to connect to others. While this definition is not a comprehensive expression of the relationship-orientation construct as it is used in the leadership literature, we believe that it can act as a suitable proxy and that it reflects at a basic level the concern for building and maintaining relationship with others.

We measure relationship-orientation by averaging the LIWC scores for two categories: personal pronouns (a proxy for self- and other-references) and social words. We were also interested about the extent to which comments contain certainty cues, and took the LIWC certainty score as the measure. Additionally, we evaluated the accuracy of an emotional category: *anger*. This is, to our knowledge, the second study (after [2]) to validate the LIWC anger category, but the first study to do so using crowdsourcing.

We crowdsourced the rating task through Crowdflower to collect assessments from a diverse base of English-speaking workers. For each of the three crowdsourced categories we selected 100 Wikipedia comments from the discussions on the article talk pages – 90 comments randomly, while 10 were selected on the basis of either highest-scoring or lowest scoring in their respective category.

We then divided the comments into pairs: comments were randomly assigned to 45 pairs, while five pairs were matched by assigning the highest scoring comment to the lowest scoring comment, and so on. The five "Gold Standard" pairs were then checked by a human rater, to ensure that the right answer is indeed obvious. A minimum of seven crowdsourced evaluators had to identify which comment from the pair is higher in relationship-orientation, certainty or anger, respectively. This matching procedure allowed us to know the correct answer for a fraction of the tasks, and therefore identify unreliable evaluators easily. Finally, we selected for analysis the ratings with a confidence level of at least 0.7, which ensured that the assessments were unambiguous.

Our results were encouraging, with accuracy levels situated around 0.70 for each of the three categories (excluding the gold standard pairs of comments). The best results were found for *certainty.* In this case 74% of assessments with LIWC coincided with those of human raters. The *anger* category achieved an accuracy level of 70%, while *relationship-orientation* came close to the 70% benchmark, with an accuracy level of 69,5%. Our results for the anger category do not depart significantly from those of Bantum and Owen [2] – they found that LIWC performs moderately well with a detection sensitivity value of 0.66, but are marginally better, which could be attributed to a higher number of raters.

We also intended to investigate a fourth category, *Cognitive Mechanisms*, which we defined as the extent to which the comment is indicative of the speaker's reflective processes, e.g. I think, I believe, and which we operationalized with the LIWC variable with the same name. Our results indicated an accuracy level of only 0.5, therefore we excluded this category from our analysis. To our knowledge, this is the first independent validation of the cognitive mechanisms category. Our validation results are quite surprising considering the research interest for this category [3, 4].

Finally, we conducted a content analysis of the comments evaluated through crowdsourcing. We were interested to see whether comments very high or very low in *relationship-orientation*, *certainty* and *anger* show distinct patterns, and whether they can be classified in different categories. This has been a successful endeavor, especially in the case of *relationship-orientation.* The insights drawn from our qualitative analyses are reported in more detail in the Results section.

Our validation work provides several methodological contributions to the rising field of automatic text analysis. First of all, we conduct a comparison of three major lexicons (LIWC, SentiStrength and ANEW), and are able to illustrate their similarities and complementarities, as well as circumstances when the lexicons converge and, respectively, diverge. All lexicons seem to have own strengths and weaknesses, and overall, they are highly complementary. Secondly, we validate several categories for the LIWC lexicons. To our knowledge, we are the first to provide validation for *relationship-orientation* and *certainty*, as well as the first to provide validation through crowdsourcing for the *anger* category. This could be valuable for researchers wanting to utilize these measures for future research.

References

1. Thelwall M, Buckley K, Paltoglou G, Cai D, Kappas A (2010) Sentiment strength detection in short informal text. J. of the American Society for Information Science and Technology 61: 2544 – 2558.

2. Bantum E, Owen J (2009) Evaluating the validity of computerized content analysis programs for identification of emotional expression in cancer narratives. Psychological Assessment 21: 79–88.

3. Pennebaker JW, Mayne TJ, Francis ME (1997) Linguistic predictors of adaptive bereavement. Journal of Personality and Social Psychology, 72: 863–871.

4. Kross E, Ayduk O (2008) Facilitating adaptive emotional analysis: Distinguishing distanced-analysis of depressive experiences from immersed-analysis and distraction. Personal and Social Psychology Bulletin 34: 924–937.
