# Supplementary material for: Emotions under Discussion: Gender, Status and Communication in Online Collaboration
Source: PLoS One. 2014 Aug 20;9(8):e104880. doi: 10.1371/journal.pone.0104880 (PMC4139304; doi:10.1371/journal.pone.0104880)
Supplement: Table S1 — Sample sizes for the Mann-Whitney tests for article and personal talk pages. (PDF) [file pone.0104880.s002.pdf]

**Table S1.** Sample sizes for the Mann-Whitney tests for article and personal talk pages.

| | Non-admins | Admins | Total |
|---|---|---|---|
| Article talk | 8 005 | 4 226 | 12 231 |
| Personal talk (written messages): LIWC | 6 644 | 4 086 | 10 730 |
| Personal talk (received messages): LIWC | 6 636 | 4 086 | 10 722 |
| Personal talk (written messages): SentiStrength | 6 198 | 3 982 | 10 180 |
| Personal talk (received messages): SentiStrength | 6 198 | 3 957 | 10 155 |
| | Males | Females | Total |
| Article talk | 2 613 | 165 | 2 778 |
| Personal talk (written messages): LIWC | 2 446 | 160 | 2 606 |
| Personal talk (received messages): LIWC | 2 445 | 160 | 2 605 |
| Personal talk (written messages): SentiStrength | 2 358 | 156 | 2 514 |
| Personal talk (received messages): SentiStrength | 2 349 | 154 | 2 503 |
